# Performance of the WHOQOL-BREF among Norwegian substance use disorder patients

**DOI:** 10.1186/s12874-019-0690-3

**Published:** 2019-03-04

**Authors:** Ashley Elizabeth Muller, Svetlana Skurtveit, Thomas Clausen

**Affiliations:** 1 Norwegian Centre for Addiction Research, Institute of Clinical Medicine, University of Oslo, Pb 1039 Blindern, 0315 Oslo, Norway; 2 Division of Health Services, Norwegian Institute of Public Health, Pb 4044 Nydalen, 0403 Oslo, Norway; 3 Department of Mental Disorders, Norwegian Institute of Public Heath, Pb 4044 Nydalen, 0403 Oslo, Norway

**Keywords:** Quality of life, Substance use disorder, Psychometrics, Social isolation

## Abstract

**Background:**

Quality of life (QoL) is an established outcome measure of substance use disorder treatment. The WHOQOL-BREF is the gold standard tool, but its appropriateness for particularly vulnerable patient populations must be further explored. This article examines the scaling qualities of the WHOQOL-BREF in a Norwegian substance use disorder population, and explores relationships with social and health variables.

**Methods:**

107 participants in a larger national treatment study provided data during structured interviews. Item responses, responsiveness, and domain scaling qualities are reported. General linear models identified correlates of impaired QoL.

**Results:**

Three out of four domains exhibited acceptable scaling qualities, while the social relationships domain had low internal validity. 59% of the variance in physical health QoL was explained in our model by the negative main or interaction effects of depression, unemployment, social isolation, smoking, residential treatment, and weight dissatisfaction. 52% of the variance in psychological health QoL was explained by depression and being single. Depression also had significant main effects in social relationships QoL (R^2^ = .27) and environment QoL (R^2^ = .39), and social isolation and exercise had further interaction effects in environment QoL.

**Conclusions:**

After one year in treatment, the impact of low social contact in reducing QoL, rather than specific substance use patterns, was striking. The social relationships domain is the shortest in the WHOQOL-BREF, yet social variables were important in other areas of QoL. Social support could benefit from more attention in treatment, as a lack of social support seems to be a strong risk factor for poor QoL in various domains. The WHOQOL-BREF exhibits otherwise satisfactory measurement characteristics and is an appropriate tool among this population.

## Background

Reducing morbidity and improving the quality of life years spent with or in recovery from a substance use disorder (SUD) is as key to treatment as reducing mortality. In the management of chronic diseases, in which the negative effects of a disease are often reoccurring and long-lasting, treatment cannot be evaluated by a cure, but by sustained improvements in self-assessed functioning and well-being. The multidimensional measure of patient-reported outcomes such as quality of life (QoL) are important outcomes of the treatment of chronic diseases such as SUDs, as it captures the repercussive impacts of disease in many areas of life that may not be identifiable through normal clinical practice [1, 2]. QoL is of further importance in SUD treatment because improving QoL may itself support treatment retention and abstinence after leaving treatment [3–5].

Reduced substance use is often assumed to automatically improve QoL. However, mixed evidence for this hypothesis among various SUD groups exists [6–9]. People may even instigate SUD treatment less to reduce their substance use than to improve their QoL in general [4, 10]. Qualitative research has shown people with SUDs consider social factors such as enough relationships and contact, a supportive network, and social inclusion as necessary components to good QoL [11–13]. Indeed, these social settings may influence the development and trajectory of SUDs in general to a far greater extent than other chronic diseases, as substance use in a person’s network impacts their own access to both substances and, later, to treatment [14–16].

Among non-SUD populations, several social determinants have been identified: Pinquart and Sorensen’s [17] meta-analysis found that the quality of social relationships among the elderly was the second most important factor in their QoL, following independence. In a cross-sectional study of people with severe mental illness, higher QoL correlated to greater social support, lower internalized and societal stigma, and lower symptom distress [18], and in longitudinal studies of people with various mental illnesses, improvements in social integration along with symptom reductions have predicted QoL improvements over time [19–21]. Sociodemographic factors, age, and gender have not been consistent predictors in these studies.

Overall QoL was reported in the aforementioned studies through single or aggregate scores [17–19, 21]. Instruments can also measure QoL multidimensionally, in which separate domains of respondents’ lives are queried and represented through separate domain scores. The gold standard in generic quality of life instruments, the World Health Organization’s brief quality of life tool (WHOQOL-BREF), measures four domains of QoL: physical health, psychological health, social relationships, and environment QoL. Strong psychometric properties have been reported internationally [22] as well as among the general populations of Nordic countries [23–25]. Normative values for this generic instrument allow for the comparison between healthy and clinical groups, such as those with SUD, and to this end, the WHOQOL-BREF has also been validated among opioid maintenance treatment patients [26–28], a substance-using veteran population [29], and most recently among an exclusively alcohol-abusing sample [30].

Two reviews have concluded that most QoL instruments administered to people with SUD under-prioritize social domains in favour of health domains, and most research on QoL determinants continues to focus on substance use and mental health [8, 31]. Two disease-specific QoL tools – the Injecting Drug Users Quality of Life Scale [32] and the Opioid Substitution Treatment Quality of Life scale [13] – both include a broader focus on social domains, but disease-specific QoL tools cannot provide QoL scores that can be compared to other populations; a recent review discusses further merits of generic measures among opioid users [33].

The WHOQOL-BREF may be a preferable generic instrument among SUD populations, given that one of its strengths is the inclusion of a social QoL domain. QoL instruments such as the WHOQOL-BREF, if they are to allow for the subjective evaluation of the effects of SUD and treatment, must also be feasible and perceived as relevant to respondents. The WHOQOL-BREF has seldom been reported for Norwegian substance users, with a small pilot study being one exception [34]. We wished to explore the acceptability of the WHOQOL-BREF to SUD patients in Norway. We analysed the WHOQOL-BREF’s item responses, responsiveness, scaling qualities, and explored the relationships of various health, substance, and social variables to each domain.

## Methods

### Participants and procedure

This paper analyses a subsample of a larger study, the Norwegian Cohort of Patients in Opioid Maintenance Treatment and other Drug Treatment study (NorComt), procedures for which have been previously described [35]. Briefly, NorComt consecutively enrolled 548 adults entering SUD treatment (either opioid maintenance treatment [OMT] or medication-free residential treatment) in 21 facilities between 2012 and 2015, with no exclusion criteria. They answered a battery of psychological and substance use questionnaires during structured interviews first when they entered treatment (baseline), by facility staff who had been trained by the research team. One year later (follow-up), the research team administered the same structured interviews to participants. There were again no inclusion criteria, and the interviews were conducted at locations chosen by participants. Sixty-two percent (*N* = 341) of the original sample at baseline was followed up with, and those lost to follow-up did not differ in terms of most baseline demographics or substance use indicators [36].

Ethical approval was granted by the Norwegian Regional Ethics Comittees (ref: 2012/1131/REK) to administer the WHOQOL-BREF to the first 100 participants consecutively interviewed at follow-up in order to assess a different instrument [37]. One hundred and seven participants ultimately answered the WHOQOL-BREF and were included in this analysis. Written informed consent was obtained from all patients before participation.

### Measures

The structured interview questionnaire was built off of the national Patient Registry Questionnaire, containing demographic variables reported by all hospitals and private contract specialists [38]. Numerous validated measures were added and are described below.

#### WHOQOL-BREF

The World Health Organization’s WHOQOL-BREF has been validated in dozens of countries and languages, and among healthy and clinical populations [22], and its appropriateness as a generic, multidimensional tool for the SUD population has been highlighted [1]. The WHOQOL-BREF produces four domain scores from 24 items: a 3-item “social relationships” domain of QoL, 7-item “physical health” domain, 6-item “psychological health” domain, and 8-item “environment domain”. Two additional items measure overall QoL and overall health. The WHOQOL-BREF uses a five-point Likert scale with answers from “very dissatisfied” to “very satisfied”, “not at all” to “an extreme amount”, and “never” to “always”. Transformed domain scores resulted in a 0–100 scale in which higher scores indicate higher QoL [39].

#### Demographic, substance, and health measures

Demographic variables included civil status (“single” or “married/partnered”), unemployment, and educational attainment of primary school or less. Substance use was measured by the substance profile from the EuropASI, which collects individuals’ four most commonly used illicit substances/unprescribed medications in the past six months [40]. Dichotomized variables represented whether a substance or medication was reported as one of a participant’s most commonly used; each participant could therefore be categorized as a user of up to four substances. Anxiety or depression warranting clinical attention were indicated by scores above 1.0 on the HSCL-25’s 10-item anxiety subscale and the 15-item depression subscale, respectively, each measured on a 0–4 scale [41]. No participant was missing more than one item per subscale, and missing items were excluded in the calculation of the mean subscales.

Those who reported at least one chronic somatic disease, e.g. coronary heart disease, diabetes, or hepatitis C, were analysed as a “chronic disease” group. Participants also self-reported smoking, using smokeless tobacco, exercising regularly in the past six months (using their own definition of exercise) and dis/satisfaction with their current weight; these variables were chosen based on associations found at baseline with low QoL [42]. Participants were categorized based on their entry at the beginning of the study into eithert OMT or medication-free residential treatment.

Finally, participants answered a social network question from the EuropASI, “with whom do you spend most of your free time?” Answers included “alone”, indicating social isolation, “with substance-using family/friends”, or “with substance-free family/friends”. This question was used as a three-category variable as well as dichotomized into a social isolation variable. Living situation was also collected in another variable and was reported as “alone”, “with partner/family”, or “with friends/others”.

### Analysis

The scaling qualities of the WHOQOL-BREF’s four domains are described, including internal validity and item-total statistics. The responsiveness of certain items in the WHOQOL-BREF to similar clinical/objective variables describing the sample are reported using Mann-Whitney U tests. To determine which of the variables collected were associated with each domain of quality of life and to what extent they could explain the domains’ variances, a general linear model was created for each domain. Domain scores were dependent variables, and independent variables were those demonstrating significant bivariate associations to each domain, tested using the Kruskal-Wallis test. HSCL-25 anxiety and depression subscores were significantly associated with each domain, and in order not to lose information in the analysis by dichotomizing them using the 1.0 cut-off score, the scores themselves were added as covariates in each adjusted model. Age was correlated with the physical health domain, and was added as a covariate only in this domain’s adjusted model. Two-way interactions were requested between all independent variables, which were included stepwise at *p* < 0.05. Model fit was reported by adjusted R^2^. All statistics were conducted in SPSS version 22.

## Results

### Participants’ characteristics

Table 1 displays the demographic, substance, health, and social variables of the 107 participants included in this analysis.

**Table 1:** Characteristics of 107 participants followed up with in the NorComt study

	n (%)
Demographics	
Age (mean, *SD*)	35.3 (*9.6*)
Women	36 (33.6)
Single	78 (77.2)
Primary education or less	61 (57.0)
Unemployed	76 (71.0)
Substance-related variables	
No substance use	27 (34.6)
Polysubstance user	49 (49.0)
Substances	
*Cannabis*	45 (42.1)
*Amphetamines*	32 (29.9)
*Alcohol*	27 (25.2)
*Unprescribed benzodiazepines*	22 (20.6)
*Heroin/opium*	19 (17.8)
*Ecstasy, LSD*	3 (2.8)
*Cocaine*	2 (1.9)
*Other central stimulants or addictive substances*	1 (0.9)
*Crack, solvents, or denatured alcohol*	0
Injected within past four weeks	25 (25.5)
Current SUD treatment	
*OMT*	46 (43.8)
*Residential*	24 (22.9)
*Outpatient (without OMT)*	15 (14.3)
*None*	20 (19.9)
Health variables	
Chronic somatic disease	71 (66.4)
Clinical anxiety symptoms	61 (57.0)
Clinical depression symptoms	60 (56.1)
Received psychiatric services, past year	55 (50.9)
Exercise	69 (64.5)
Dissatisfied with weight	53 (49.5)
Smoke cigarettes	90 (84.1)
Use smokeless tobacco	36 (34.3)
Social variables	
Social network	
*Abstinent*	59 (55.1)
*Substance-using*	28 (26.2)
*No network*	20 (18.7)
Living situation	
*With partner/family*	33 (30.8)
*Friends or others*	13 (12.1)
*Alone*	61 (57.0)

*SUD* substance use disorder

*OMT* Opioid maintenance treatment

Participants followed up with in this sub-study were a majority men, single, and unemployed. Most lived alone, had an abstinent social network, and exhibited symptoms of clinical anxiety or depression

34% of participants were women, and the mean age was 34.3 (*SD* = 9.6). The majority were single, had a primary education or lower, and were unemployed. Sixty-five participants had entered into OMT (60.7%) and 42 had begun residential treatment (39.3%) at study inclusion. When interviewed again at follow-up, 19.9% were no longer in treatment, 22.9% were in residential treatment, 43.8% were in OMT, and the 14.3% were receiving non-OMT outpatient treatment. Half (49.0%) reported polysubstance use in the past 6 months, while 19.6% reported only one substance, and 34.6% reported none (excluding prescribed medication users). Participants reported their four most frequently used substances in this time period from an exhaustive list, and cannabis was the most common (reported by 42.1%), followed by amphetamines (29.9%), alcohol (25.2%), unprescribed benzodiazepines (20.6%), and heroin (17.8%). One fourth (25.5%) had injected within the past 4 weeks.

Two-thirds reported at least one chronic somatic disease (66.4%), while nearly 60% reported symptoms of clinical anxiety or clinical depression, and half had received professional psychiatric services in the past year. Half were dissatisfied with their weight. While most smoked cigarettes (84.1%) and one third used smokeless tobacco (34.3%), exercising was also common (64.5%). Finally, 18.7% reported being socially isolated, while half (55.5%) said their social network was abstinent, and 26.2% had a substance-using network. Most participants lived alone (57.0%), one third with a partner or family (30.8%), and 12.1% with friends or others.

### WHOQOL-BREF item responses and responsiveness

There were few skipped or missing data overall in the WHOQOL-BREF. 99.8% of the items were answered; only five participants (4.6%) had any missing data, and each participant skipped no more than one item. The item “how satisfied are you with your sex life?” was missing for four of these participants, all of whom reported being single. The fifth participant did not answer the item “how satisfied are you with the support you get from your friends?”, although did not report being isolated.

No ceiling or floor effects were identified for any of the items. The most skewed item was in the physical health QoL domain, “to what extent do you feel that physical pain prevents you from doing what you need to do?” 51% of respondents selected the most impaired response, “an extreme amount”, and in decreasing proportions towards the least impaired response, “not at all”, selected by only 4%. Another item in this domain, “How much do you need any medical treatment to function in your daily life?” had a U-shape, with nearly equal proportions answering the two extremes: 28% “not at all” and 26% “an extreme amount”.

The responsiveness of several items in the WHOQOL-BREF to convergent objective variables was promising. The item, “How much do you need any medical treatment to function in your daily life?” successfully distinguished between those currently in opioid maintenance treatment and those not, *p* < 0.001. Similarly, “How often do you have negative feelings such as blue mood, despair, anxiety, depression?” distinguished between those with symptoms of clinical depression (*p* < 0.001), clinical anxiety (*p* < 0.001), and who had received psychiatric services in the past year (*p* = 0.014). Unemployment status was distinguished by responses to the item, “How satisfied are you with your capacity for work?” (*p* < 0.001). “Are you able to accept your bodily appearance?” distinguished between those were satisfied with their weight versus dissatisfied (*p* = 0.017).

Finally, two items are included in the WHOQOL-BREF which do not contribute to domain scores. An item asking for an overall rating of QoL was answered “very poor” by 0.9%, “poor” by 13.1%, “neither good nor poor” by 34.6%, “good” by 36.4%, and “very good” by 15.0%. In response to “how satisfied are you with your health?”, 2.8% answered “very dissatisfied”, 18.7% “dissatisfied”, 31.8% “neither satisfied nor dissatisfied”, “37.4%” “satisfied”, and 9.3% “very satisfied”.

### Scaling qualities

Table 2 displays scaling qualities of the WHOQOL-BREF. The physical health domain had the lowest mean (45) and median (46) as well as the smallest range (4–82). The social relationships domain was scored the highest (mean: 62, median: 58), a larger range (8–100), and the most normal and symmetric distribution among the domains. Psychological health and environment domains had similar means (55 and 57, respectively).

**Table 2:** Scaling qualities of the WHOQOL-BREF domains

	Physical health QoL (*N* = 107)	Psychological health QoL (*N* = 107)	Social relation-ships QoL (*N* = 102)	Environment QoL (*N* = 107)
Mean (*SD*)	44.87 (16.98)	54.60 (17.11)	62.34 (18.50)	57.29 (16.82)
Median (IQR)	46.43 (17.83–17.03)	54.41 (33.57–75.25)	58.33 (33.33–83.33)	56.25 (33.60–78.90)
Range	3.6–82.1	16.67–100	8.33–100	25–96.88
CI	41.59–48.10	51.32–57.87	58.70–65.97	54.06–60.51
Kurtosis (SE)	−0.27 (0.46)	0.26 (0.46)	0.14 (0.47)	−0.73 (0.46)
Skewness (SE)	−0.29 (0.23)	0.25 (0.23)	−0.19 (0.24)	0.16 (0.23)
Significant test of normality^a^	*p* = 0.037	*p* = 0.004	*p* < 0.001	*p* = 0.024
Cronbach’s alpha	0.763	0.792	0.541	0.762

^a^One-Sample Kolmogorov-Smirnov Tests. *QoL* quality of life, *IQR* interquartile range, *SE* standard error

Physical health, psychological health, and environment QoL domains of the WHOQOL-BREF exhibited satisfactory scaling qualities among a substance use disorder treatment population

Cronbach’s alpha was acceptable for the physical health QoL (0.763), psychological health QoL (0.792), and environment QoL (0.762), but lower for social relationships QoL, (0.541). Reliability analysis suggested all items were worthy of retention. All items had acceptable corrected item-total correlation, greater than 0.3 and up to 0.7, with their respective domain scores. The four domain scores correlated moderately to strongly with one another, with correlation coefficients, displayed in Table 3.

**Table 3:** WHOQOL-BREF domain correlations

	Physical health	Psychological health	Social relationships
Psychological health	.533^a^		
Social relationships	.506^a^	.553^a^	
Environment	.553^a^	.503^a^	.472^a^

Numbers are Sphearman’s rho. ^a^Correlation is significant at the 0.01 level (2-tailed)

The four domains of the WHOQOL-BREF correlated moderately to strongly with one another

### Correlates of impaired QoL

#### Physical health QoL domain

The following groups differed in their physical health QoL scores, on a 0–100 scale (*p* < 0.05): participants who were unemployed (40.5), smoked (43.4), physically inactive (36.1), and socially isolated (32.1) reported lower scores than those who were employed (55.6), non-smokers (52.7), physically active (49.7), and were not socially isolated (47.1), respectively. In addition, participants who were dissatisfied with their weight reported worse physical health QoL (39.9) than those who evaluated their weight as appropriate (49.7). Lower scores were also reported by those who used benzodiazepines (40.3) than those who did not (47.7), and by participants in the OMT cohort (42.9) instead of residential treatment (57.1).

In an adjusted model containing these variables (Table 2), depression and unemployment had significant main effects on physical health QoL. An increase in the HSCL-25 depression score and unemployment reduced this domain by 8.0 points, and unemployment reduced this domain score by 26.9 points. Social isolation did not have a significant main effect, but several interaction effects including social isolation did. The interaction between social isolation and smoking lowered one’s score by 32.4 points. The interaction between social isolation and entering the study in residential treatment as opposed to OMT lowered this score by 20.2 points, and the interaction between social isolation and weight dissatisfaction lowered this score by 16.6 points. This model accounted for 59.0% of the variance.

#### Psychological health QoL domain

Bivariate analyses showed that participants who were partnered or married instead of single reported higher psychological health QoL (60.4 compared to 52.1), as well as those who lived with a partner or family (60.3) instead of alone (52.2) or with friends or others (51.5).

In an adjusted model with these variables (Table 4), living situation has no main effect or interaction effect, but there were significant main effects of depression and being single. An increase in the HSCL-25 depression score corresponds to a decrease in 11.9 points in psychological health QoL, and being single instead of partnered/married reduces this score by 9.4 points. This model explains 52.4% of the variance.

**Table 4:** Adjusted models explaining variance in WHOQOL-BREF domains

Variables with significant main effects or interaction effects	ß	*p*	R^2^
Physical health QoL^a^			0.590
Depression score	−8.0	0.001	
Unemployment	−26.9	0.048	
Social isolation * smoking	−32.4	0.040	
Social isolation * residential treatment	−20.2	0.034	
Social isolation * weight dissatisfaction	−16.6	0.026	
Psychological health QoL^b^			0.524
Depression score	−11.9	< 0.001	
Single	−9.4	0.037	
Social relationships QoL^c^			0.271
Depression score	−14.3	0.001	
Environment QoL^d^			0.386
Depression score	−7.2	0.043	
Social isolation * no cannabis	−27.0	0.036	
Social isolation * exercise	−19.6	0.004	
Exercise * no amphetamines	+ 26.8	0.003	

*QoL* Quality of life

Not significant in adjusted models, after stepwise inclusion: ^a^unemployment, smoking, physical inactivity, weight dissatisfaction, benzodiazepine use, opioid maintenance treatment cohort. ^b^partner status, living situation. ^c^social isolation. ^d^employment, psychiatric services received, social isolation, chronic disease, physical activity, polysubstance use, cannabis use, amphetamine use

Depression and social factors, rather than the direct effects of substance use or severity, explained most of the variance of the physical health and psychological health QoL domains of the WHOQOL-BREF

#### Social relationships QoL domain

Those who reported social isolation reported lower social relationships QoL (54.8) than those not isolated (64.1). This was the only variable with a significant correlation to social relationships QoL.

In the adjusted model (Table 4), only depression had a significant main effect on domain scores. An increase in depression scores resulted in a 14-point decrease in social relationships QoL domain. There was also a trend for a negative effect of social isolation. This model explains only 27.1% of the combined variance of QoL scores.

#### Environment QoL domain

The environment QoL domain had the most variables with significant bivariate associations: lower environment QoL was reported by those who were unemployed (53.8) compared to employed (65.7), had not received psychiatric services in the past year (54.0) compared to those who had (60.4), were socially isolated (46.2) instead of having a network (59.8), had a chronic disease (54.7) instead of none (62.4), were physically inactive (50.0) instead of active (60.6), were polysubstance users (50.2) compared to single-substance users (60.8) or substance-free/prescribed-only substance users (64.6), consumed cannabis (51.8) instead of no cannabis (61.2), and consumed amphetamines (46.8) instead of no amphetamines (61.8).

In the adjusted model (Table 4), an increase in the HSCL-25 depression score reduced the environment QoL domain by 7.2 points. There was also an interaction effect of exercising and not consuming amphetamines, improving environment QoL by 26.8 points. Social isolation did not have a significant main effect, but had a negative interaction effect with several other variables. The interaction effect of being socially isolated and not consuming cannabis reduced this domain score by 27.0 points, while being socially isolated and exercising reduced this domains core by 19.6 points. There was also a trend for the interaction effect of social isolation and having a chronic disease to decrease environment QoL. This model accounted for 38.6% of the variance of environment QoL.

## Discussion

In the first such analysis of the WHOQOL-BREF in Norway, lacking regular (reported) social contact had a strong negative effect on numerous dimensions of QoL of 107 participants in a substance use disorder treatment study. Along with depression, social factors were more important than direct effects of substance use or of severity indicators. The WHOQOL-BREF exhibited satisfactory scaling qualities and user acceptability and is therefore overall considered a useful tool among this population, but its social relationships domain needs further investigation. We recommend that the social lives of people with SUD receive more attention in future QoL instrument development and in treatment.

The poor QoL reported by this sample is in line with a larger body of research on SUD populations [7, 43, 44]. Of novelty in this analysis is our exploration of social contributors to QoL. This sample reported concerningly low levels of social contact and engagement, with 18% reporting being isolated, 57% living alone, 77% without a partner, and 71% disconnected from both the labour market and educational system. A qualitative study of OMT patients in Belgium reported a similar paucity of social contact, and patients linked their lack of daily activities to feeling alone and without emotional support [45]. The effects of such social disengagement in our sample extended beyond the social relationships QoL domain, and low social contact had negative interaction effects in every other domain. Social isolation appears so detrimental to well-being that it overrode the positive effect of exercising in the environment QoL domain. The environment domain may also indicate social inclusion, as it measures aspects such as access to social care and participation in leisure activities. This sample reported slightly lower environment QoL than social relationships QoL.

Other negative health-related variables such as smoking and being dissatisfied with one’s weight, in tandem with social isolation, reduced the physical health QoL domain. Smoking and weight dissatisfaction are common among people in SUD treatment [46–48], which is particularly concerning given evidence that smoking cessation is associated with higher rates of long-term abstinence from other substances [49], yet is not standardly integrated into treatment programs [50]. More than half of the sample reported symptoms of clinical depression, which was a component of poorer QoL in every domain, in alignment with extensive international [7, 8, 51], Scandinavian [46], and Norwegian research [52] reporting mental health as a factor in QoL among SUD populations. We found no correlation between active substance use or treatment variables to the physical health, psychological health or social relationships domains of QoL, contributing to existing mixed evidence of these relationships among people with SUD [6–9, 53, 54]. The environment QoL domain was most responsive to substance use variables in bivariate analyses and to a lesser extent in the adjusted model, and the validity of this domain may be higher for people in active substance-using phases than for those in other populations.

Many of the components of poor QoL seen in this analysis can be addressed in treatment. When depression is addressed, symptoms can improve significantly and their corresponding treatment barriers alleviated [55]. Similarly, lifestyle interventions among SUD populations have been successfully implemented to reduce smoking and encourage other health-promoting behaviours such as healthy eating, sleep hygiene, and exercise [46, 56–58]. Social contact itself can support healthy behavioural change, as reported in a recent qualitative analysis of alcohol treatment patients who dropped out of an exercise intervention [59]. These results suggest that in addition to mental health and exercise, specific interventions aiming at improving social contact should also be a focus during treatment.

Social isolation was operationalized through answering the question, “with whom do you spend most of your free time?” with “mostly alone”. Patients may have responded differently had they been asked directly if they were isolated. Being mostly alone could be a temporary situation resulting from a recent change in social situation, such as loss of a job or a partner, or following relocation. It could also be an intentional technique utilized by those who would otherwise only have substance-using people around them, as described in previous qualitative studies [60–62] At the same time, people in recovery have also reported loneliness as an instigator for relapse [63, 64]. As Mau et al. [65] have recently discussed, while the agency of people with SUD in negotiating their own loneliness and isolation should be acknowledged, “loneliness should not be clinically accepted as a requirement or side-effect of recovery in the long-term” (p.12).

The WHOQOL-BREF performed well as a whole, with the exception of the social relationships domain. Expected social variables such as isolation or relationship status did not explain observed variance in this domain, and in fact, our model did not adequately explain variance. Most likely, the three questions that comprise this domain – how satisfied are you with your personal relationships, support from friends, and sex life? – are simply too few, and should be supplemented with items from the larger WHOQOL-100 instrument. The negative interaction effects of low social contact on almost all domains, along with the low internal consistency of the social relationships domain, underscore the need for QoL tools to capture the social lives of people with SUD and likely other marginalised groups, but the importance of social factors is belied by the size of this domain in the WHOQOL-BREF. This may require the development of a new generic QoL tool with a greater focus on social QoL.

The heterogeneity of this sample – including users of different substances and having entered different treatment modalities – is a strength of this study, as the SUD population itself is diverse. Nevertheless, this sample was relatively small, prohibiting stratified analyses such as by gender. No gender differences in QoL were found in previous analyses from the larger study from which this sample was drawn [42, 66], but there may be a gender aspect to social networks and contact, and it is worth exploring this further. Another limitation is that the larger study did not utilize a random sampling design, limiting generalizability of the study’s and of this sample’s characteristics to the Norwegian patient population. While this analysis is therefore less capable of providing specific prevalence rates, the correlations found between QoL, exercise, and social contact seem stable, as they were also found in analysis of the larger study at baseline [42] and follow-up [66].

## Conclusion

The WHOQOL-BREF is a short, valid measure of QoL among a SUD treatment population and exhibited acceptable scaling properties and item discrimination. More attention to the social relationships domain is required, however, and better information may be collected through additional questions/developments. The negative effect of low social contact with other variables was seen in every domain of QoL, and treatment providers should monitor the contact patients report when they enter treatment, during treatment, and at discharge. Addressing the quality and functionality of the social contact and support that patients have may be an important part of intake and future treatment processes, in order to best support patients’ engagement with people supportive to their recovery. These findings may also be relevant for other patient groups with high rates of isolation, and a focus on increasing social contact may be an important element to add to the health care of marginalized populations.

## Abbreviations

NorComt: Norwegian Cohort of Patients in Opioid Maintenance Treatment and other Drug Treatment study
QoL: Quality of life
SUD: Substance use disorder
WHOQOL-BREF: World Health Organization’s Quality of Life Brief assessment
